# A mental health training program for community health workers in India: impact on knowledge and attitudes

**DOI:** 10.1186/1752-4458-5-17

**Published:** 2011-08-05

**Authors:** Gregory Armstrong, Michelle Kermode, Shoba Raja, Sujatha Suja, Prabha Chandra, Anthony F Jorm

**Affiliations:** 1 Nossal Institute for Global Health, University of Melbourne, Level 4, Alan Gilbert Building, 161 Barry St, Carlton 3010, Australia; 2 Basic Needs, No. 114, 4th Cross OMBR Layout, Bengaluru 560043, India; 3 National Institute for Mental Health and Neurosciences (NIMHANS), Hosur Road, Hombegowda Nagar, Bengaluru 560029, India; 4 Orygen Youth Health Research Centre, Centre for Youth Mental Health, University of Melbourne, Locked Bag 10, Parkville 3052, Australia

## Abstract

**Background:**

Unmet needs for mental health treatment in low income countries are pervasive. If mental health is to be effectively integrated into primary health care in low income countries like India then grass-roots workers need to acquire relevant knowledge and skills to be able to recognise, refer and support people experiencing mental disorders in their own communities. This study aims to provide a mental health training intervention to community health workers in Bangalore Rural District, Karnataka, India, and to evaluate the impact of this training on mental health literacy.

**Methods:**

A pre-test post-test study design was undertaken with assessment of mental health literacy at three time points; baseline, completion of the training, and three month follow-up. Mental health literacy was assessed using the interviewer-administered Mental Health Literacy Survey. The training intervention was a four day course based on a facilitator's manual developed specifically for community health workers in India.

**Results:**

70 community health workers from Doddaballapur, Bangalore Rural District were recuited for the study. The training course improved participants' ability to recognize a mental disorder in a vignette, and reduced participants' faith in unhelpful and potentially harmful pharmacological interventions. There was evidence of a minor reduction in stigmatizing attitudes, and it was unclear if the training resulted in a change in participants' faith in recovery following treatment.

**Conclusion:**

The findings from this study indicate that the training course demonstrated potential to be an effective way to improve some aspects of mental health literacy, and highlights strategies for strengthening the training course.

## Background

Mental disorders are both disabling and costly for affected individuals, their families and the community. Mental disorders are increasingly recognised as a major contributor to the global health burden, including in low income countries (LICs), and they are often co-morbid with communicable and non-communicable diseases [1,2]. Mental health remains a low priority in most LICs, and unmet needs for mental health treatment are pervasive [3-5]. Up to 90% of persons with mental disorders in low and middle-income countries do not receive even basic mental health care [4,6]. This neglect continues despite overwhelming evidence that effective low-cost treatments (drugs, psychological treatments, and community based rehabilitation) are feasible, affordable and cost-effective for many mental disorders, and could be successfully delivered in primary health care (PHC) settings [1,7-9].

In India, approximately 6% of the population have a mental disorder such as schizophrenia [7]. Suicide is a major public health problem, with over 100,000 suicides annually [10]. Mental disorders in India are not necessarily experienced and understood in the same way as in Western countries [11-15], and the vast majority of care is provided by the family. Many remain untreated, and those families who do seek treatment will often turn to non-allopathic providers including practitioners of Indian traditional medicine, religious healers, faith healers and astrologers [13,14,16]. The scarcity of mental health professionals, particularly in rural areas, places specialist psychiatric care out of the reach of most people [7,10,17].

The World Health Organization (WHO) advocates the need to integration of mental health into PHC as the optimal strategy for addressing the global burden of disease [7,8]. In India, the National Mental Health Program also advocates the integration of mental health into PHC; however, there has been limited success in realising this policy in practice with only 24 of 600 districts currently covered by this program [7,10,18]. Due to the overlap between mental and physical health, people with mental disorders frequently present to PHC settings. For example, a survey in a Mumbai slum found that 28% of patients aged > 18 years attending a health centre suffered from psychiatric problems [12]. However, PHC staff lack the skills required to make an appropriate diagnosis and provide a reasonable standard of care when people present with such problems. Effective training programs are required to develop the mental health skills of generalist PHC staff [7].

If mental health is to be successfully integrated into PHC in low-income countries like India then grass-roots workers need to acquire relevant knowledge and skills so that they are able to recognise, refer and support people experiencing mental health disorders in their own communities. Task shifting of effective mental health interventions to non-specialist health workers has been proposed to increase the coverage of mental health care in both low and high-income settings [19-25].

Mental Health Literacy (MHL) is defined as 'knowledge and beliefs about mental disorders which aid their recognition, management and prevention' (p.396) [26]. Studies in India have indicated that knowledge and understanding of mental disorders is poor in many communities, including among community health workers [27-29]. Improved awareness of mental disorders among community health workers is likely to assist affected people to access treatment and improve the quality of the care they receive [26,30]. Having the knowledge and skills to support people in the community who may be developing a mental disorder or experiencing a mental health crisis is referred to as Mental Health First Aid [31].

A small number of studies investigating various components of integrating mental health into PHC settings in India, have been conducted [26,32-36] and this is creating an opportunity and momentum for achieving real changes in practice, leading to better outcomes for people with mental disorders. This study makes a contribution to this emerging body of evidence as it evaluates the effectiveness of a mental health training manual for grass-roots community health workers (CHWs). Mental health training was delivered to CHWs who had minimal knowledge about mental health, and the impact of the training on their level of MHL was evaluated. The hypothesis of the study was that community health workers would demonstrate improved MHL as assessed at completion of the training program and three months later.

## Methods

### Study design

This study involved an evaluation of a mental health training program using a pre-test post-test design. Assessment of participants' MHL was undertaken at baseline, at completion of the course and three months later. The training and data collection was conducted between May and October 2010. Ethics approval was obtained from the University of Melbourne Health Sciences Human Ethics Sub-Committee.

### Participants and facilitators

Training participants were community health workers sourced through Gramina Abrudaya Seva Samstha (GASS), an NGO operating in Doddaballapur Taluk, Bangalore Rural District, Karnataka, India. The types of community health workers included Junior Health Assistants, Village Rehabilitation Workers, and ASHA workers. These are all categories of government-funded community health workers operating in Doddaballapur Taluk, that each have a range of tasks to undertake from the provision of maternal and child health care, to building community awareness about communicable and non-communicable diseases, to disability rehabilitation. The participants were trained in three separate groups of between 23 and 24 participants by the same two facilitators who were both local health professionals with a moderate level of experience and understanding of community mental health. The level of experience of the facilitators reflected a 'real world' scenario given that experienced mental health professionals are rare in rural settings in India.

### The intervention

The mental health training program is a four-day course that aims to increase recognition of mental disorders, enhance appropriate response and referral, support people with mental disorders and their families, and improve mental health promotion in communities. It is not intended as a training program for mental health practitioners, but rather as an introduction to mental health for uninitiated community health workers. The training program is based on a facilitator's manual [37] developed by some of the study investigators, the design and content of which was informed by the literature [7,8,38-42]. The content of the training program includes an introduction to mental health and mental disorders, mental health first aid, practice-based skills, and mental health promotion (detailed in Table 1). The facilitator's manual is designed to provide: 1) a plan for each training session including the purpose, timing and required materials, 2) background information for each session, 3) a series of case studies that provide realistic scenarios describing people possibly experiencing mental disorders, 4) suggestions for participatory activities and role-plays, and 5) images and diagrams to assist in explaining concepts and frameworks.

**Table 1:** Content of introduction to mental health training program for community health workers

(1) **An introduction to mental health and disorders**	➢ *Factors affecting mental health* ➢ *Symptoms of mental disorders* ➢ *Severe and common mental disorders*
(2) Mental health first aid	➢ *Responding to people with;* ■ *Unexplained physical complaints* ■ *Excess worry and panic* ■ *Unusual sadness or suicidal thoughts* ■ *Persistent tiredness* ■ *Sleeping problems* ■ *Psychotic symptoms*
(3) **Practice-based skills**	➢ *Introduction to counselling* ➢ *Problem solving techniques* ➢ *Home visits* ➢ *Supporting the family* ➢ *Referring to mental health professionals* ➢ *Understanding drug treatments*

(4) **Mental health promotion**	➢ *Introduction to mental health promotion* ➢ *Stigma and discrimination* ➢ *Poverty and mental health* ➢ *Gender and mental health*

### Questionnaire design and outcome measures

**Figure 1:**
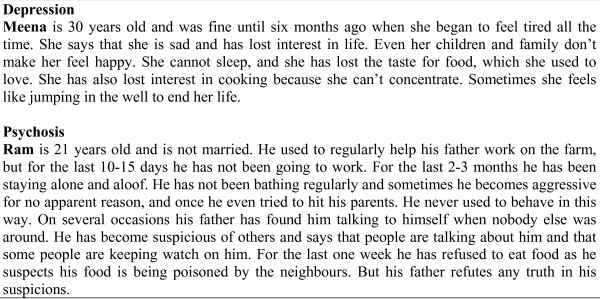
**Vignettes for depression and psychosis from the MHL survey**.

To assess changes in the participants' level of MHL, we adapted a MHL survey previously used in rural India [27-29] and in Australia [42,43], including with migrant communities [44,45]. This MHL survey involves presentation of two vignettes, each describing people experiencing symptoms of mental disorders (depression, psychosis) (Figure 1). Using a combination of open-ended and structured questions, participants were asked to identify the problems, their causes, and effective sources of help. They were also asked about attitudes towards people with mental disorders, and anticipated outcomes for them. Only responses to structured questions with pre-coded response options are reported on in this paper. The MHL survey was administered face-to-face by trained interviewers (due to limited literacy of some participants), and took about 30 minutes to complete. Each participant was matched to the same interviewer at each point of measurement.

To ensure the MHL survey was appropriately translated for the local setting, the English version was reviewed with local psychiatrists, and the relevance of concepts and categories and the appropriate form of translation into the local language (Kannada) were extensively discussed. The survey was then translated and back-translated into English to check for equivalence of meaning, and subsequently piloted.

### Sample size

Based on a change in the percentage of respondents who were able to correctly recognise a mental disorder in a vignette, we estimated that we would require a total of 63 training participants to detect a medium effect size (Cohen's h = 0.5, approximately a 20-25% difference) with a power of 80%, alpha of 0.05, and making the conservative assumption of no correlation between pre-test and post-test [46]. The sample size of 70 was chosen based on both the power analysis and feasibility, since this was the maximum possible given the time and financial constraints, and would result in training being provided to the majority of government-funded community health workers operating in Doddaballapur Taluk, Bangalore Rural District.

### Analysis

Statistical analysis was performed with SPSS version 18. Dichotomous variables were analysed using the Cochran's Q test to test for variation between the three points of measurement; baseline, post-course, and three month follow-up. McNemar's test for two paired proportions was used to specifically examine changes between baseline and post-course, and between baseline and three month follow-up. All tests were performed separately for responses to questions about each of the two vignettes (i.e. depression and psychosis) in the MHL questionnaire. Respondents who didn't complete all three points of measurement were omitted from the analysis.

## Results

### Participant characteristics

There were 70 participants recruited for the study and only one participant did not complete the training. A further three participants were not available at the three month follow-up leaving 66 (94.3%) participants who had completed the MHL survey at all three points of measurement. Table 2 presents the characteristics of these 66 study participants. The mean age of participants was 37 years, with a range of 21 to 59 years. The majority were female (86.4%) and married (75.8%). Approximately half the participants (46.9%) had not completed high school (i.e. 12 years of education). More than half of the participants (66.7%) were Junior Health Assistants, 24.2% were Village Rehabilitation Workers (VRWs), and 9.1% were Asha workers.

**Table 2:** Characteristics of participants (n = 66)

Characteristic	n (%)
***Age (years)***	

≤25	15 (22.7)

26-35	20 (30.3)

36-45	16 (24.2)

≥46	15 (22.7)

***Gender***	

Female	57 (86.4)

Male	9 (13.6)

***Education***	

1 to 4	3 (4.5)

5 to 11	28 (42.4)

12	33 (50.0)

Tertiary	2 (3.0)

***Marital status***	

Single	12 (18.2)

Married	50 (75.8)

Widowed	3 (4.5)

Divorced	1 (1.5)

***Job title***	

Junior health assistant	44 (66.7)

Village rehab worker	16 (24.2)

Asha worker	6 (9.1)

### Recognition of disorders in vignettes

Participants were read the two vignettes and asked to name the problem (more than one response was possible). Only "depression", "schizophrenia" or "psychosis" were considered correct responses to the relevant vignettes. A substantial improvement in the participants' ability to recognise mental disorders was observed at the completion of the training and sustained for the three-month follow-up assessment (Table 3). Prior to receiving the training program, few respondents (9.1%) were able to correctly recognise the disorder in the psychosis vignette, and less than a quarter (22.7%) were able to correctly recognise the disorder in the depression vignette. There was a statistically significant increase in the percentage of participants able to correctly identify depression and psychosis after receiving the training intervention. There was a drop in the percentage of participants who could correctly identify depression between post-course and follow-up measurements, however, the difference between baseline and follow-up (22.7% to 43.9%) was found to be statistically significant (McNemar's test, p = 0.022).

**Table 3:** Change in the recognition of depression and psychosis

	Pre-course % (95% C.I.)	Post-course % (95% C.I.)	3 Month Follow-up % (95% C.I.)	p-value*
**Depression**	22.7 (13.3-24.7)	50.0 (37.4-62.7)	43.9 (31.7-56.7)	0.002

**Psychosis**	9.1 (3.4-18.7)	27.3 (17.0-39.6)	34.8 (23.5-47.6)	0.001

* Cochran Q-test.

### Perceived helpfulness of interventions

Participants were asked about the helpfulness or otherwise of a range of possible pharmacological and non-pharmacological interventions for the problems identified in the vignettes (Table 4). Regarding pharmacological interventions, there was a sustained decrease in the percentage of participants endorsing potentially useless pharmacological interventions such as vitamins including tonics and herbal medicines, appetite stimulants, and sleeping pills, and a sustained but small increase in those endorsing other pharmacological treatments.

**Table 4:** Percentage of participants identifying various interventions as helpful

	Pre-course % (95% C.I.)	Post-course % (95% C.I.)	3 Month Follow-up % (95% C.I.)	p-value*
***Pharmacological interventions***				

**Depression**				

Vitamins, tonics or herbal medicines	71.2 (58.7-81.7)	45.5 (33.1-58.2)	36.4 (24.9-49.1)	< 0.001

Appetite stimulants	69.7 (57.1-80.4)	34.8 (23.5-47.6)	30.3 (19.6-42.9)	< 0.001

Sleeping pills	42.4 (30.3-55.2)	19.7 (10.9-31.3)	4.5 (0.9-12.7)	< 0.001

Other pharmacological	12.1 (5.4-22.5)	19.7 (10.9-31.3)	28.8 (18.3-41.3)	0.058

**Psychosis**				

Vitamins	74.2 (62.0-84.2)	48.5 (36.0-61.1)	31.8 (20.9-44.4)	< 0.001

Appetite stimulants	65.2 (52.4-76.5)	39.4 (27.6-52.2)	30.3 (19.6-42.9)	< 0.001

Sleeping pills	47.0 (34.6-59.7)	21.2 (12.1-33.0)	4.5 (0.9-12.7)	< 0.001

Other pharmacological	7.6 (2.5-16.8)	18.2 (9.7-29.6)	34.8 (23.5-47.6)	0.001

***Non-pharmacological interventions***				

**Depression**				

Physical activity	92.4 (83.2-97.5)	95.5 (87.3-99.1)	92.4 (83.2-97.5)	0.670

Distraction	98.5 (91.8-100.0)	98.5 (91.8-99.9)	100.0 (94.6-100.0)	0.607

Love and affection	100.0 (94.7-100.0)	100.0 (94.6-100.0)	100.0 (94.6-100.0)	1.000

Listening	95.5 (87.3-99.1)	100.0 (94.6-100.0)	100.0 (94.6-100.0)	0.050

Hospital admission	60.6 (47.8-72.4)	69.7 (57.1-80.4)	43.9 (31.7-56.7)	0.003

Special diet	15.2 (7.5-26.1)	9.1 (3.4-18.7)	6.1 (1.6-14.8)	0.155

**Psychosis**				

Physical activity	95.5 (87.3-99.1)	97.0 (89.5-99.6)	95.5 (87.3-99.1)	0.867

Distraction	98.5 (91.8-100.0)	98.5 (91.8-100.0)	100.0 (94.6-100.0)	0.607

Love and affection	100.0 (94.6-100.0)	100.0 (94.6-100.0)	100.0 (94.6-100.0)	1.000

Listening	97.0 (89.5-99.6)	100.0 (94.6-100.0)	100.0 (94.6-100.0)	0.135

Hospital admission	61.5 (48.6-73.3)	66.2 (53.4-77.4)	51.5 (38.9-64.0)	0.182

Special diet	19.7 (10.9-31.3)	6.1 (1.7-14.8)	4.5 (0.9-12.7)	0.006

Getting person married**	72.7 (60.4-83.0)	29.7 18.9-42.4)	65.2 (52.4-76.5)	< 0.001

* Cochran Q-test ** This response option was only available with the psychosis vignette - the person in the depression vignette was already married.

There were no clear and sustained changes in the endorsement of various non-pharmacological interventions, other than a decrease in the percentage of participants endorsing a special diet as helpful for the psychosis vignette. At baseline, psychosocial interventions such as physical activity, distraction, love and affection and listening were strongly endorsed as being helpful for both vignettes, and this was sustained across both points of follow-up.

There was a statistically significant change in the percentage of participants endorsing hospital admission as a helpful response for the depression vignette between the three points of measurement, however, the greatest difference was between post-course and follow-up measurement. The decreased endorsement of hospital admission for the depression vignette between baseline and follow-up (60.6% to 43.9%) was not statistically significant (McNemar's test, p = 0.063).

There was a substantial reduction in the percentage of participants endorsing marriage as a helpful intervention for the person in the psychosis vignette between baseline and post-course measurement (McNemar's test, p = < 0.001), however this change was not sustained at the three month follow-up. The decrease in the endorsement of marriage for the psychosis vignette between baseline and follow-up (72.7% to 65.2%) was not statistically significant (McNemar's test, p = 0.424).

### Perceived outcomes for people with mental disorders

Participants were asked structured questions about the anticipated recovery of the persons described in the vignettes if appropriate help is received (Table 5). For the depression vignette, there was no statistically significant change in anticipated prognosis. There was a statistically significant reduction in the percentage of participants identifying 'full recovery, no further problems' for the person described in the psychosis vignette. At baseline, the majority of participants reported that the persons in both the depression (95.5%) and psychosis (92.4%) vignettes would get worse if no appropriate help was given, and this remained largely unchanged at follow-up.

**Table 5:** Percentage identifying likely outcomes if help is received

	Pre-course % (95% C.I.)	Post-course % (95% C.I.)	3 Month Follow-up % (95% C.I.)	p-value*
**Depression**				

Full recovery, no further problems	36.4 (24.9-49.1)	36.4 (24.8-49.1)	24.2 (14.5-36.4)	0.210

Full recovery, problems probably re-occur	22.7 (13.3-34.7)	27.3 (17.0-39.6)	34.8 (23.5-47.6)	0.247

Partial recovery	18.2 (9.8-29.6)	9.1 (3.4-18.7)	18.2 (9.8-29.6)	0.209

Partial recovery, problems probably re-occur	22.7 (13.3-34.7)	27.3 (17.0-39.6)	22.7 (13.3-34.7)	0.767

**Psychosis**				

Full recovery, no further problems	47.0 (34.6-59.7)	40.9 (29.0-53.7)	19.7 (10.9-31.3)	0.003

Full recovery, problems probably re-occur	10.6 (4.4-20.6)	18.2 (9.8-29.6)	22.7 (13.3-34.7)	0.152

Partial recovery	21.2 (12.1-33.0)	6.1 (1.7-14.8)	18.2 (9.8-29.6)	0.030

Partial recovery, problems probably re-occur	22.1 (12.1-33.0)	34.8 (23.5-47.6)	37.9 (26.2-50.7)	0.107

* Cochran Q-test.

### Attitudes to people with mental disorders

Participants were asked whether they agreed with a range of attitudinal statements relating to the persons described in the vignettes, and while some improvements in attitudes were observed, others remained largely unchanged (Table 6). For both the depression and the psychosis vignettes at baseline, a majority of participants agreed that the person could 'snap out of it', that the problem was a sign of personal weakness, and that people with this problem are erratic. Additionally, a significant minority perceived the persons described in the vignettes as dangerous and approximately half said they would not vote for people with these problems, and this did not change after receiving the training.

**Table 6:** Percentage agreeing to various statements about the persons in the vignettes

	Depression			
	
	Pre-course % (95% C.I.)	Post-course % (95% C.I.)	3 Month Follow-up % (95% C.I.)	p-value*
**People with this problem can snap out of it**	64.6 (51.8-76.1)	56.1 (43.3-68.3)	53.0 (40.3-65.4)	0.281

**This problem is a sign of personal weakness**	84.8 (73.9-92.5)	89.4 (79.4-95.6)	62.1 (49.3-73.8)	< 0.001

**This problem is not a real medical illness**	54.5 (41.8-66.9)	51.5 (38.9-64.0)	59.1 (46.3-71.0)	0.636

**People with this problem are dangerous**	30.8 (19.9-43.4)	37.9 (26.2-50.7)	33.3 (22.2-46.0)	0.717

**It is best to avoid people with this problem**	21.2 (12.1-33.0)	6.1 (1.7-14.8)	4.5 (0.9-12.7)	0.001

**People with this problem are erratic**	77.3 (65.3-86.7)	90.9 (81.3-96.7)	80.3 (68.7-89.1)	0.054

**I would not vote for a person with this problem**	50.0 (37.4-62.6)	54.5 (41.8-66.9)	53.0 (40.3-65.4)	0.836

	**Psychosis**			
	
	**Pre-course %** **(95% C.I.)**	**Post-course %** **(95% C.I.)**	**3 Month Follow-up %** **(95% C.I.)**	p-value*

**People with this problem can snap out of it**	67.2 (54.3-78.4)	42.4 (30.3-55.2)	51.5 (38.9-64.0)	0.002

**This problem is a sign of personal weakness**	74.2 (62.0-84.2)	86.4 (75.7-93.6)	63.6 (50.9-75.1)	0.004

**This problem is not a real medical illness**	48.5 (36.0-61.1)	40.9 (29.0-53.7)	63.6 (50.9-75.1)	0.017

**People with this problem are dangerous**	44.6 (32.3-57.5)	40.9 (29.0-53.7)	45.5 (33.1-58.2)	0.828

**It is best to avoid people with this problem**	18.2 (9.8-29.6)	7.6 (2.5-16.8)	15.2 (7.5-26.1)	0.128

**People with this problem are erratic**	80.3 (68.7-89.1)	92.4 (83.2-97.5)	84.8 (73.9-92.5)	0.097

**I would not vote for a person with this problem**	48.5 (36.0-61.1)	60.6 (47.8-72.4)	57.6 (44.8-69.7)	0.217

* Cochran Q-test.

With respect to the depression vignette, the training resulted in a marked decrease in the percentage agreeing that it is best to avoid people with these problems. Additionally, there was a reduction between baseline and follow-up (84.8% to 62.1%) in the percentage who agreed that the problems described in the depression vignette were a sign of personal weakness (McNemar's Test, p = 0.009); however, there was no statistically significant change between baseline and post-course measurement (McNemar's Test, p = 0.057).

In the case of the psychosis vignette, there were no sustained changes in responses to the attitudinal statements that could be clearly attributed to the training intervention. Fewer participants agreed that the person could 'snap out of it' at post-course (42.4%) measurement than at baseline (67.2%) (McNemar's Test, p = 0.002), however the decrease between baseline and follow-up (51.5%) was not statistically significant (McNemar's Test, p = 0.089) indicating that the improved attitude had not been sustained. The Cochran's Q test indicated a statistically significant change in the percentage of participants agreeing that the problem is a sign of personal weakness; however, this was due to the change between the results at post-course and follow-up measurements. There was no statistically significant change in the proportion agreeing with this statement between baseline and post-course measurement (McNemar's test, p = 0.057) or between baseline and follow-up (McNemar's test, p = 0.210).

At baseline, approximately half the participants agreed that the problems described in both the depression and psychosis vignettes were not real medical illnesses. There was no real change in this belief in the case of the depression vignette, but there was some statistically significant variation over time in relation to psychosis as identified by the Cochran's Q test. However, this was due to the changes between the results at post-course and follow-up measurements. There was no statistically significant change in the proportion agreeing with this statement between baseline and post-course measurement (McNemar's test, p = 0.458) or between baseline and follow-up (McNemar's test, p = 0.100).

## Discussion

The issue of providing practical mental health training for primary health care workers in low-income countries is of broad interest to many working in the field of global mental health. This study makes an important contribution to the related literature by conducting an evaluation of a training program for community health workers in rural India using an existing facilitator's mental health training manual [37].

The findings suggest that the mental health training program had mixed success in achieving its stated aims, but there were some encouraging outcomes. Importantly, there was only one participant out of seventy who did not complete the training program despite the extensive travel required for many participants to attend, and the considerable demands of participants' daily working and living duties.

The training increased the ability of the community health workers to recognize depression and psychosis in vignettes, and reduced their faith in unhelpful pharmacological interventions. There was evidence of a minor reduction in stigmatising attitudes, although the changes were very limited and largely isolated to the depression vignette. It was unclear if the training resulted in a change in the participant's faith in recovery.

The percentage of respondents correctly identifying the disorders in the vignettes in the baseline questionnaire was low relative to a previous survey of village health workers in a rural area of Maharashtra, India (depression, 56.7%) [28], and much lower than a sample of Australian community members trained in Mental Health First Aid (depression, 91.4%; psychosis 56.6%) [42]. The results suggest that the training increased the ability of the participants to recognise depression and psychosis in vignettes, yet despite this improvement, more than half of the participants were still unable to correctly identify either disorder at the three month follow-up. However, an evaluation of a mental health first aid training program conducted in Australia (delivered by an experienced mental health instructor) with a community sample of immigrants of Chinese-speaking background found results comparable to our study; recognition of depression and psychosis increased from 19.0% to 63.1% and 9.5% to 21.4% respectively immediately after the training (no three month follow-up was conducted) [44].

At baseline, psychosocial interventions, including physical activity and listening, were widely viewed as helpful by participants, and there was moderately high endorsement of potentially inappropriate treatments like appetite stimulants, vitamins, tonics, herbal medicines, and sleeping pills. These findings are comparable to those from the rural Maharashtran MHL survey mentioned above [28]. The significant decrease in the endorsement of inappropriate pharmacological treatments among training participants is an important finding given that, whilst vitamins, tonics and herbal medicines may be relatively harmless, appetite stimulants and sleeping pills are of doubtful value and in some cases may be harmful. Similarly, the drop in endorsement for special diets is desirable given the lack of good evidence to support this as an intervention for depression or psychosis [47]. While psychiatric medications were not asked about explicitly, the increasing endorsement of the "other pharmacological" category suggests an improvement in awareness of appropriate psychiatric medication options that have an important role to play in recovery, particularly from severe mental illness.

Less than half the participants believed that, with appropriate help, the persons described in the vignettes could fully recover without further problems. Additionally, belief in full recovery with no further problems decreased in the case of the psychosis vignette, however, it appeared that the significant decrease happened after the training (i.e. between post-course measurement and three month follow-up) making it difficult to directly attribute this change to the training intervention itself. In any case, these negative outcome expectations are concerning and have the potential to impede access to potentially effective treatments. The result may also reflect the local context of the study setting where there are no local psychiatrists or highly skilled mental health practitioners. Additionally, the training intervention itself did not focus on evidence for the effectiveness of treatments for mental disorders. This is an important consideration given that there is evidence indicating that belief in the effectiveness of treatments can help to reduce stigmatising attitudes towards people with mental disorders [48,49].

The training had a small impact on stigmatising attitudes towards people with mental disorders. Perhaps most importantly, after participation in the training fewer participants thought that the mental disorder described in the depression vignette was a sign of personal weakness, and only a small percentage agreed that it is best to avoid the person described in this vignette.

The concepts of stigma and discrimination in the context of mental disorders have been well documented [50,51]. Stigma is linked to experiences of discrimination by people with mental disorders [52], including biases in the receipt of healthcare [53]. Stigma (as expressed by mental health professionals and the general public) contributes to under-use of mental health services, a delay in accessing mental health treatment, and an impeded recovery process [48,54-56]. The negative effects of stigma can outweigh the impact of disability due to the disorder [57].

Culture influences the experience, expression and determinants of stigma, and the effectiveness of different approaches to stigma reduction, and further research on attitudes toward mental illness is required in India and other non-Western cultures. Results from a study on patients with schizophrenia and their caregivers in Bangalore [58] indicated that stigma motivated families to contain the patient at home in an effort to conceal the condition and the perceived causes of the condition (e.g. sins and bad deeds), resulting in a delay in receiving timely treatment. Families and patients experiencing a high level of stigma would attempt to avoid the social disapproval anticipated from seeking allopathic care.

The content of the mental health training manual in this study incorporated a brief educational activity regarding the topic of stigma and discrimination that was focused on exploring the impact of stigma and identifying some common stigmatising attitudes. Our findings indicate that the effectiveness of the training in relation to stigma reduction was limited, and that it would be worthwhile exploring opportunities to have a greater impact in this area.

Research has highlighted the importance of contact with people experiencing mental disorders as a strategy for changing stigmatising attitudes about mental illness [51,59-65]. A study using a community sample from America [66] found that direct contact with people who have (or have had) mental health problems had the greatest impact on attitudes, in comparison to education challenging myths which yielded only a modest positive impact. Involving people who have previously experienced mental disorders as co-facilitators has the potential to reduce stigmatising attitudes of participants, as does contact with consumers who have had positive (and realistic) experiences of mental health care, and who are employed and socially included in their communities [48]. While the training manual evaluated in this study can be modified to more actively promote the inclusion of consumers as facilitators/presenters, it must be acknowledged that there are still substantial barriers to be overcome in order to consistently achieve this. In a country such as India, people with mental disorders most frequently remain hidden and are not mobilised precisely because of the stigma experienced by affected individuals and families.

A second useful strategy for improving stigmatising attitudes as identified by Corrigan et al [48] is to provide more information about the effectiveness of treatments. There is an association between confidence in treatment and stigmatising attitudes [49]. This approach could be complemented by discussions that explore approaches for building trust in allopathic mental health care within the community, and increasing contact with people with mental disorders so that participants can be exposed to positive consumer stories about recovery.

### Limitations

There are several limitations to this study, including the lack of a control group. Whilst there is no obvious reason for knowledge and attitudes to change over time without having had the training, it is plausible that repeated testing alone may produce some change or that the participants gave certain responses to please the researchers due to a social desirability bias. It is also possible that a relevant event occurred in the general community (e.g. a story in the media about a person with a mental disorder) in-between points of measurement that may have caused participants to change their responses to the questions in the MHL survey. The lack of a control group makes it difficult to confidently explain some of the changes in participant's responses, particularly those changes between post-course measurement and three month follow-up. However, we believe the pre-test post-test design was appropriate for this evaluation study, and acknowledge that further research in this area would benefit from using a randomised controlled trial design with a control group who could be wait-listed to receive the training after data collection was completed.

There are potential limitations to the cultural transportability of the MHL questionnaire. Despite careful and consultative adaptation, translation and piloting of the training program as well as the MHL questionnaire, we must be cautious in presuming an equivalence of meaning across cultures and languages. Certainly explanatory models of mental health and illness are culturally determined and therefore not equivalent across cultures, and this may effect the utility of the MHL survey, even though it was locally adapted. Subtleties of meaning and cultural factors may have influenced the way in which the facilitators interpreted the content of the training manual, the messages taken away from the training by the participants, and the interpretation of the questions in the MHL survey.

A comparison of findings between this study and similar evaluations of mental health training conducted elsewhere should be interpreted with caution. Firstly, the community health worker participants had minimal knowledge of mental health as explained by the Western model of psychiatry. Terms such as depression and schizophrenia do not easily translate linguistically or culturally. Secondly, rather than using facilitators who were very experienced in the field of mental health, our study sought to replicate a "real world" situation involving facilitators who were local community health leaders and who had minimal prior experience in delivering mental health training of this nature. They were required to work with a manual written in English and translate complex concepts in a coherent way, whilst managing group conversations about the cross-cultural understanding of the material. The outcomes may well have been different if the facilitators were more experienced in both the field of mental health and the effective use of active-learning methods of training as designed in this manual.

Finally, the training in this study was provided to participants in four consecutive days for logistical reasons, despite the recommendation in the training manual that it be spread out. Ideally, participants would have time to reflect on the material in-between sessions, particularly given that the material was new for most participants and complicated by the cultural issues that arise when discussing mental health in India.

The impact of the training on the self-confidence of the community health workers, the rates of mental health consultations and referrals, and benefits for people experiencing mental health problems including improvements in pathways to treatment and recovery, remain unknown. To achieve substantial improvements in community mental health, it is likely that a comprehensive suite of interventions including community awareness programs and improved integration of mental health into primary health care is required.

## Conclusion

The mental health training facilitator's manual demonstrated potential to be an effective way to improve some aspects of mental health literacy among community health workers, including the recognition of mental disorders in vignettes and the perceived helpfulness of inappropriate pharmacological interventions. The training had a limited positive impact on attitudes towards people with mental disorders. Strategies for improving the training could include facilitating contact with consumers of mental health services, including stories of recovery, and providing information on the effectiveness of interventions for mental disorders.

## Competing interests

The authors declare that they have no competing interests.

## Authors' contributions

All authors contributed to interpretation of the findings and development of the manuscript. GA and SS coordinated the study implementation. GA and MK undertook the statistical analysis and wrote the first draft of the manuscript. SR and MK assisted with the study implementation. PC provided expertise on the local context of mental health and reviewed the questionnaire for appropriate translation of key concepts. AFJ designed the original mental health literacy questionnaire that was adapted for this study, and provided expertise on study design and data analysis. All authors read and approved the final manuscript.
